# The effects of venlafaxine on depressive-like behaviors and gut microbiome in cuprizone-treated mice

**DOI:** 10.3389/fpsyt.2024.1347867

**Published:** 2024-06-03

**Authors:** Chunhai Du, Tian Zhang, Chong Feng, Qian Sun, ZhiGuo Chen, Xin Shen, Ying Liu, Gengwu Dai, Xuan Zhang, Nailong Tang

**Affiliations:** ^1^ Department of Oncology, Hengshui Hospital of Traditional Chinese Medicine, Hengshui, Hebei, China; ^2^ Department of Psychiatry, Xijing Hospital, Air Force Medical University, Xi’an, Shaanxi, China; ^3^ Department of Psychiatry, The 907th Hospital of the PLA Joint Logistics Support Force, Nanping, Fujian, China; ^4^ Institute for Hospital Management Research, Chinese PLA General Hospital, Beijing, China

**Keywords:** depressive-like behavior, cuprizone, gut microbiota, venlafaxine, depression

## Abstract

**Background:**

Cuprizone (CPZ)-treated mice show significant demyelination, altered gut microbiome, and depressive-like behaviors. However, the effects of venlafaxine (Ven) on the gut microbiome and depressive-like behavior of CPZ-treated mice are largely unclear.

**Methods:**

Male C57BL/6J mice were fed a chow containing 0.2% cuprizone (w/w) for 5 weeks to induce a model of demyelination. Meanwhile, the gut microbiota and depressive-like behaviors were assessed after the mice were fed with Ven (20 mg/kg/day) or equal volumes of distilled water for 2 weeks by oral gavage from the third week onward during CPZ treatment.

**Results:**

CPZ treatment decreased the sucrose preference rate in the sucrose preference test and increased the immobility time in the tail-suspension test, and it also induced an abnormality in β-diversity and changes in microbial composition. Ven alleviated the depressive-like behavior and regulated the composition of the gut microbiota, such as the increase of *Lactobacillus* and *Bifidobacterium* in CPZ-treated mice.

**Conclusion:**

The anti-depressant effects of Ven might be related to the regulation of gut microbiota in the CPZ-treated mice.

## 1 Introduction

Major depressive disorder (MDD) is the most prevalent mental illness that affects millions of individuals and constitutes a substantial share of the disease burden globally (1). The lifetime prevalence of depression ranges from 20% to 25% in women and from 7% to 12% in men (2, 3). Besides the persistent loss of interest and sadness, MDD is always accompanied by appetite, disturbed sleep, low self-worth, tiredness, and hopelessness. MDD also leads to nearly 800,000 people dying of suicide every year (4). The causative factors of MDD are complex and may be related to multiple factors such as genetic, hormonal, neuroimmune, environmental, and psychological stress (5). Compared with healthy subjects, patients with MDD showed reductions in myelin content, axon numbers, and myelin-related genes and proteins in various brain areas (6, 7). Consistently, decreased white matter (WM) volume and total length of myelinated fibers in WM were observed in a chronic unpredictable stress (CUS) model of depression in rats (8), and a reduction in oligodendrocyte-related proteins and myelin were also shown in a mice model of unpredictable chronic mild stress (UCMS) (9). Moreover, impaired WM integrity is more common in other neurological disorders that are accompanied by depressive symptoms, such as multiple sclerosis (MS) (10) and Parkinson’s disease (11). Myelin damage might participate in the pathogenesis of MDD, and the disruption of proliferation and differentiation of oligodendrocytes (OL) lineage cell may contribute to the depression phenotype (12–14).

There is growing evidence that MDD may be closely related to gut microbiota abnormalities and that the gut microbiota can affect the central nervous system through the gut–brain axis, altering gut–brain signaling in patients, which, in turn, plays a crucial role in host physiology, homeostasis, development and metabolism (15, 16). Patients with MDD exhibit disruptions in the gut microbiota and its metabolites (17, 18), and alterations in gut microbiota composition are also a hallmark of depression (19, 20). Meanwhile, prebiotics and probiotics showed anti-depressive effects (21, 22). In turn, dysfunction in the gut microbiota induces depressive-like behaviors and reinforces the risk of depression (23–26)—for example, when the gut microbiota of a depressed-like mice is transplanted into a normal mouse gavaged with antibiotics, that mouse exhibits a depression-like behavior (23, 27, 28). In contrast, transplantation of healthy population microbiota into mice with depressive-like behavior attenuates depressive-like symptoms (29, 30). Recently, studies further showed that *Dialister* and *Coprococcus* were found to be depleted in MDD patients (31, 32) and indicated a potential role of microbial γ-aminobutyric acid (GABA) production in depression (33). Intriguingly, gut microbiota has also been associated with processes such as myelin formation and development. Studies have shown that gut microbiota-derived metabolites and cellular components are important to improve brain homeostasis and the progression of neuropsychological problems—for example, tryptophan precursors and metabolites, 5-hydroxytryptamine (5-HT), GABA, glutamine, histamine, branched-chain amino acids (BCAAs), LPS, and catecholamines are important host/microbial-derived metabolites or components that occur in the regulation of neurogenesis, glial cell function, and myelin formation (34, 35). In the experimental autoimmune encephalomyelitis (EAE) mouse model, altered gut microbes also affect myelin integrity, with mice treated with broad-spectrum antibiotics preventing motor dysfunction and myelin damage, while bacterial recolonization impairs motor function and axonal integrity (36, 37). In short, there is a potential link between myelin integrity and specific alterations in the gut microbiota.

Venlafaxine (Ven) is a 5-HT and norepinephrine reuptake inhibitor (SNRI) antidepressant that has been widely used in MDD, anxiety disorders, and neuropathy. Among them, 5-HT plays a neurotransmitter role in the brain and enteric nervous system, participates in mood and cognitive regulation, and also regulates gastrointestinal secretion and motility in the enteric nervous system (38, 39). Treatment with Ven at a dosage of 10 mg/kg/day has been demonstrated to effectively reverse the reductions in body weight, motor activity, and sucrose consumption induced by chronic unpredictable mild stress (CUMS) and ameliorate depressive-like behaviors (40). Additionally, Ven has been found to attenuate myelin loss in the corpus callosum and prefrontal cortex, leading to an increase in the total number of oligodendrocyte lineage (OL) cells, including non-maturing oligodendrocytes (41). These findings highlight the significant role of venlafaxine in protecting the myelin, although the precise mechanism underlying this protective effect remains unclear. Meanwhile, a recent work found that Ven decreases the abundance but not the homogeneity of the microbial community and regulates the composition of the microorganisms, with a significant decrease in the relative abundance of *Ruminococcus* and *Adlercreutzia*, which are associated with its antidepressant effects (42). However, the effects of Ven on depressive-like behavior and regulation of gut microbial structure and function in a mouse model of demyelination have not been fully characterized.

Bis-cyclohexanone-oxaldihydrazone (cuprizone, CPZ) is a copper chelator that induces loss of myelin in specific brain regions when fed to rodents (43). The widely used CPZ model was established by Hiremath and colleagues in 1998, which used lower-dose CPZ (0.2% w/w) administration in C57BL/6 mice (44). CPZ is commonly administered for 4–6 weeks to study acute demyelination and to study remyelination after CPZ withdrawal. Although it was the most commonly used MS model, previous studies have confirmed that CPZ-treated mice exhibit significant depressive-like behaviors (41, 45). Moreover, CPZ administration also leads to an abnormal composition of the gut microbiota (37, 46). Considering the above-mentioned details, the purpose of this study was to explore the effects of Ven on the intestinal microbiome and depressive-like effects of CPZ model mice. The data could shed more light on the effects of antidepressants on gut microbes.

## 2 Materials and methods

### 2.1 Experimental design

**Figure 1:**
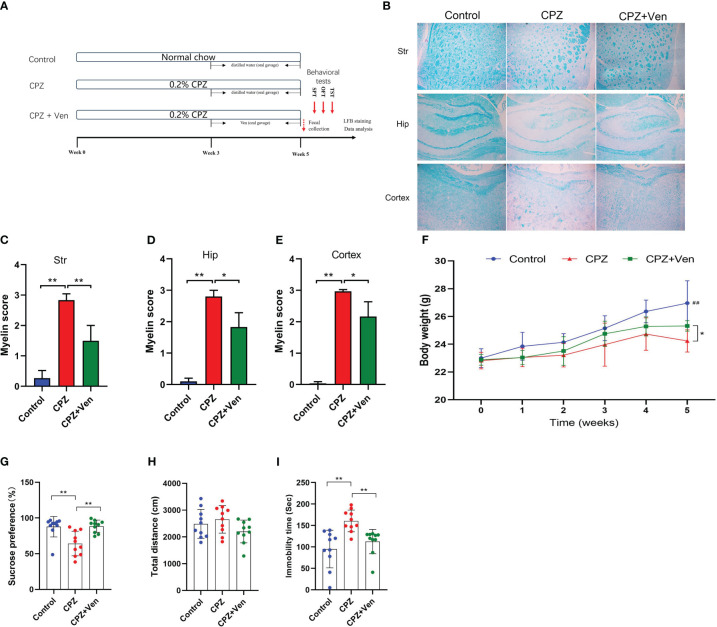
Ven improves cuprizone (CPZ)-induced depressive-like behaviors. **(A)** Experimental timeline. **(B)** FBL staining showed the myelin staining in the stritum (Str), hippocampus (Hip) and cerebral cortex in the three groups. **(C–E)** Quantitative analysis of demyelination was performed by measuring the percentage of immunostained area using ImageJ in the **(C)** stritum (Str), **(D)** hippocampus (Hip), and **(E)** cerebral cortex in the three groups. Data are presented as mean ± SD. **(F)** Changes in body weight in control, CPZ, and CPZ + Ven groups. ^##^ *P* < 0.01 vs. CPZ or CPZ + Ven group. **(G)** Sucrose preference rate in SPT test, **(H)** total distance traveled in the OPT, and **(I)** immobility time in the TST test. The circle represents one value from individual mice in control, CPZ, and CPZ + Ven groups, respectively. *P < 0.05; **P < 0.01.

To investigate the impact of venlafaxine (Ven) on the gut microbiome of CPZ-treated mice, 30 mice were randomly divided into three groups (10 per group)—control, CPZ, and CPZ + Ven—after 1 week of acclimatization (**Figure 1A**). The mice in the control group received regular chows plus daily treatment for 5 weeks. The mice in CPZ and CPZ + Ven groups were administered with CPZ (Sigma-Aldrich, St. Louis, USA), 0.2% by weight, in standard powdered rodent chow for 5 weeks (47, 48). From the third week onward, the CPZ + Ven group was fed with venlafaxine at 20 mg/kg/day for 2 weeks by oral gavage, whereas the control and CPZ groups were fed with equal volumes of distilled water. Body weight was measured at each weekend. Behavioral tests were performed to evaluate depressive-like behaviors during the sixth week. Finally, the mice were sacrificed, and the brains were obtained for fast blue staining. Data analysis was conducted by experimenters who were blind to the experimental grouping.

### 2.2 Animals

Adult male C57BL/6 mice (8 weeks old, 18–22 g in weight) were group-housed (*n* = 5) in cages (310 × 205 × 180 mm) at 20°C–25°C and maintained on a 12-h light/dark daily cycle (lights on from 8 a.m. to 8 p.m.) with unrestricted access to food and water. All animal experiments in this study were approved by the Animal Experimental Welfare & Ethical Inspection of Air Force Medical University (20230452) and carried out in accordance with the National Institutes of Health Guide for the Care and Use of Laboratory Animals.

### 2.3 Fecal sample

Fecal samples were collected when the mice were placed in a metabolic cage between 7:00 a.m. and 11:00 a.m. on the first day after the completion of the modeling. Defecation in undefecated mice was promoted by lifting their tails, ensuring that at least one fecal sample was collected from each mouse at each collection time point. The fecal samples were collected in sterile cryotubes and immediately frozen in liquid nitrogen before further analysis.

### 2.4 Behavioral tests

Before the formal test of all behavioral experiments, the mice were put into the experimental environment for half an hour in advance. The tests were videotaped and scored by a trained observer. The sucrose preference test (SPT) was conducted prior to the open-field test (OFT), and the tail suspension test (TST) was performed 24 h after the OFT. The test area was cleaned with 75% ethanol between tests to prevent odor interference and cross-infection.

#### 2.4.1 SPT

Prior to the SPT test, the mice were group-housed in cages and underwent a 1% sucrose water adaptive training for 48 h. First, the mice were adapted to two bottles of 1% sucrose solution for 24 h, and then a bottle of 1% sucrose solution was replaced with pure water for another 24 h. After the adaptive training, the mice were deprived of water and food for 24 h. At the beginning of the formal experiment, all mice were individually placed in cages, and each mouse was given two bottles of quantitative liquid: a bottle of 1% sucrose solution and a bottle of pure water. The positions of the bottles were switched every 1 h. The consumption of sucrose solution and pure water was weighed 2 h later, and the sucrose preference rate was calculated: sucrose preference rate (%) = sucrose consumption/(sucrose consumption + water consumption).

#### 2.4.2 OFT

The OFT was performed according to a recent study (49). The mice were placed in the center of an open-field box (40 cm × 40 cm × 40 cm), and then the activity was recorded for a period of 5 min. The total distance traveled was measured by using the activity software (Top Scan, Clever Sys Inc., USA).

#### 2.4.3 TST

After having the mouse adapted to the environment, the tail of the mouse was fixed with adhesive tape and hung on the test rack (40 cm above the ground) for 6 min. Then, the mouse was returned to the cage, and the tape was gently removed to prevent additional pain. Their activity for the duration of the last 5 min was analyzed, and immobility was defined as lack of skeletal movement for at least 1 s (analyzed by Top Scan behavioral analysis software from side view).

### 2.5 Histopathological examination

The mice were anesthetized (sodium pentobarbital, i.p. 50 mg/kg) and then perfused with 4% paraformaldehyde in phosphate-buffered saline (PBS). The brains were removed and transferred to 30% sucrose in PBS for 1 week to dehydrate and then sectioned (15-μm brain coronal sections) with a cryostat and mounted on gelatinized slides. Myelination was examined by Luxol Fast blue (LFB, Sigma-Aldrich) staining. After the slices were cleaned with distilled water, they were immersed in 0.1% LFB solution, sealed at 60°C for 8–16 h, and continued to be separated by 0.05% lithium carbonate solution for more than 10 s. Then, the color separation was continued with 70% alcohol until the gray and white matter were clearly distinguished under the microscope. The neutral resin seal was photographed for microscopic observations, and the myelin sheath was bright blue.

Demyelination was measured using an improved semi-quantitative scale system (0–3), with a score of 0 indicating normal myelin status, 1 indicating only one-third of the myelin bundle fibers equivalent to demyelination, 2 indicating two-thirds demyelination, and 3 indicating complete myelin degradation (50). The scores of the different parts were added together to find the average score for each group.

### 2.6 Gut microbiota profiling by 16S rRNA gene sequencing

To profile the microbial composition, fecal samples were subjected to total genome DNA extraction using the HiPure Stool DNA Kit following the manufacturer’s instruction (D3141, Guang Zhou Genedenovo, China). The V3–V4 region of 16S rRNA genes of the samples was amplified by polymerase chain reaction (PCR) (98°C for 60 s, followed by 30 cycles at 98°C for 10 s, 50°C for 30 s, 72°C for 60 s, and 72°C for 5 min) using primers 341F 5′-CCTACGGGNGGCWGCAG-3′ and 806R 5′-GGACTACHVGGGTATCTAAT907–3′. The sequencing libraries of the V3–V4 region of the 16 S rRNA genes were generated using the TruSeq^®^ DNA PCR-Free Sample Preparation Kit (Illumina, San Diego, CA, USA) following the manufacturer’s instruction, and index codes were added. Libraries were sequenced using an Illumina Novaseq 6000 sequencing platform. Bioinformatic analysis was performed using Omicsmart, a real-time interactive online platform for data analysis (http://www.omicsmart.com).

### 2.7 Statistical analysis

Upon 16S rRNA gene sequencing, 9,680,987 sequences were generated for high-quality filtering and chimera examination, with an average length of 456.38 bp for tags across all samples. The average number of reads per sample was 122,047, ranging from 36,252 to 137,595 clean reads. Based on relative abundances, the taxonomic analysis suggested 12 bacterial phyla, 19 classes, 37 orders, 55 families, and 120 genera [based on species abundances >0.1% count, in which low abundance species/operational taxonomic units (OTUs) have been filtered out]. Body weight, behavioral outcomes (total distance traveled, sucrose preference index, and immobility time), and the quantification of demyelination were assessed and compared between the groups using one-way analysis of variance (ANOVA). The results are expressed as mean ± SEM. *p* < 0.05 was considered statistically significant. OTUs with 97% similarity were used for α and β diversity statistics. The PCoA analysis was based on the Bray–Curtis index, and weighted and unweighted UniFrac metrics were used to assess changes in bacterial composition between groups and stages. LEfSe analysis, combined with Kruskal–Wallis test and linear discriminant analysis, was used to identify differences between the three groups of fecal microbiome (linear discriminant analysis, LDA >3.0]. The data were tested for normality, potentially followed by an independent sample *t*-test or a non-parametric test depending on the test results. SPSS 22.0 (IBM, Armonk, USA) and GraphPad Prism software (version 8.0) were used for statistical analysis.

## 3 Results

### 3.1 Ven improves CPZ-induced myelin injury and depressive-like behaviors

The effect of Ven on myelin injury induced by CPZ was studied by LFB staining of brain sections. As shown in **Figures 1B–E**, 5 weeks of CPZ treatment caused a significant myelin damage in the striatum, hippocampus, and cerebral cortex of the mice. The results of LFB staining showed that, compared with the control group, the staining of striatum (*F*_2, 6_ = 41.58, *P* < 0.001), hippocampus (*F*_2, 6_ = 66.49, *P* < 0.001), and cerebral cortex (*F*_2, 6_ = 89.97, *P* < 0.001) decreased. The demyelination score was significantly higher (**Figures 1C–E**), and demyelination was partially alleviated by Ven intervention (CPZ vs. CPZ + Ven, *P* < 0.05). Meanwhile, the body weight of mice in the CPZ group and CPZ + Ven group was lower than that in the control group after 5 weeks of CPZ feeding (*P* < 0.01), and Ven treatment increased the body weight of CPZ-treated mice (CPZ vs. CPZ + Ven, *P* < 0.05, **Figure 1F**). There were also significant differences in the sucrose preference rate (*F*_2, 27_ = 9.948, *P* < 0.01, **Figure 1G**) and immobility time in TST (*F*_2, 27_ = 10.35, *P* < 0.01, **Figure 1I**). However, there was no significant difference in the total distance traveled in the OPT (*F*_2, 27_ = 2.108, *P* = 0.141, **Figure 1H**) and total liquid intake (*F*_2, 27_ = 0.495, *P* = 0.617) among the three group. *Post hoc* comparisons further showed that CPZ reduced the sucrose preference rate but increased the immobility time (CPZ vs. Control, *P* < 0.01). Ven ameliorated the depressive-like behavior induced by CPZ, as evidenced by the increasing sucrose preference rate and decreasing immobility time observed in the Ven + CPZ group (CPZ vs. CPZ + Ven, *P* < 0.01).

### 3.2 Ven influences β-diversity and the composition of gut microbiota in CPZ-treated mice

**Figure 2:**
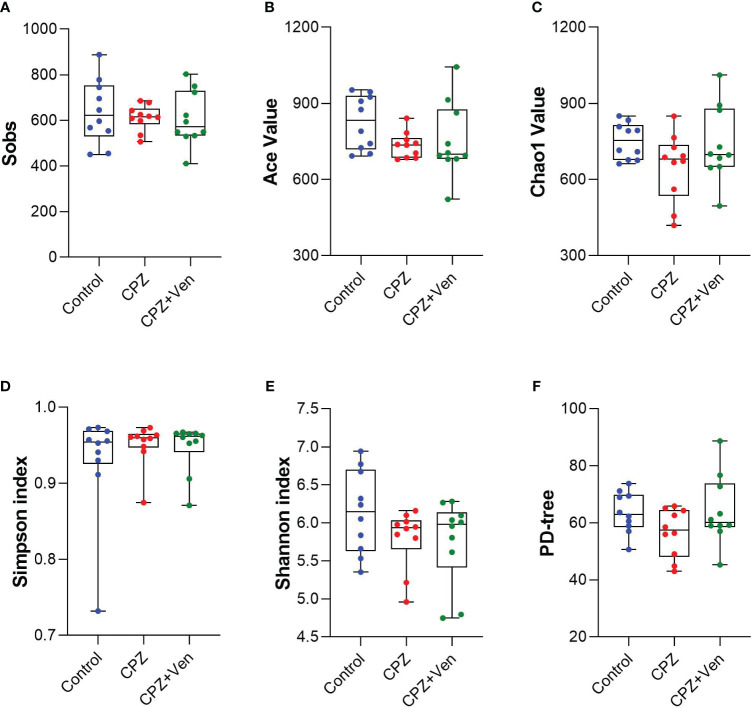
Comparison of α-diversity of gut microbiota among control, cuprizone (CPZ), and CPZ + Ven groups. The box plots depicted the indices of **(A)** Sobs, **(B)** Ace value, **(C)** Chao1 value, **(D)** Simpson and **(E)** Shannon index, and **(F)** PD tree of the operational taxonomic unit level. The horizontal lines in the box plots represent median values; the upper and lower ranges of the box represent the 75% and 25% quartiles, respectively. The blue, red, and green circle represents one value from individual mice in each group, respectively.

**Figure 3:**
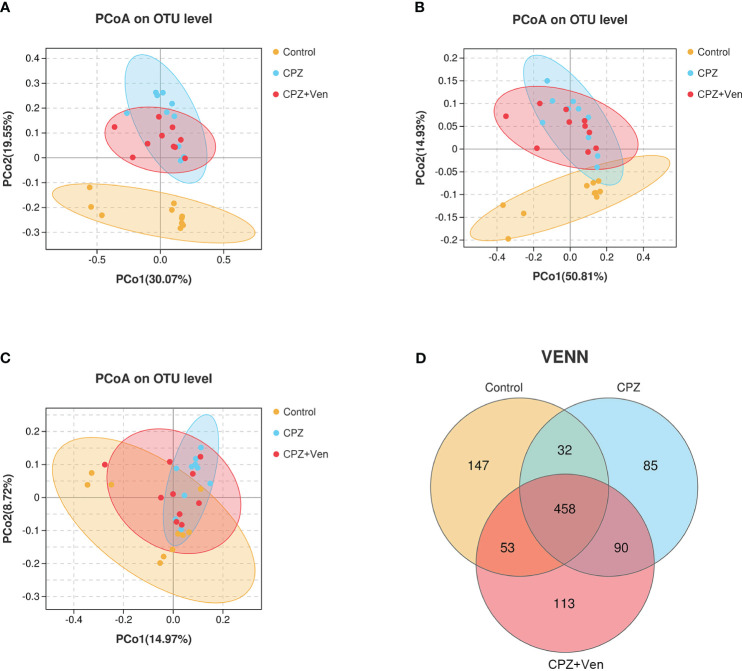
Comparison of β-diversity among control, cuprizone (CPZ), and CPZ + Ven groups. PCoA plots of bacterial beta-diversity based on **(A)** Bray–Curtis, **(B)** weighted UniFrac, and **(C)** unweighted UniFrac distance. **(D)** The number of common and unique operational taxonomic units between each group is displayed by the Venn diagram. The yellow, blue, or red circle represents one value from individual mice in control, CPZ, and CPZ + Ven group, respectively.

To assess the overall differences in microbial community structure between the control, CPZ, and CPZ + Ven groups, we calculated α- and β-diversity measures for ecological diversity within and between the given samples. There were no significant differences in comparative α-diversity among the three groups, including Sobs, ACE, Chao1, Simpson, Shannon, and PD tree (*P* > 0.05, **Figures 2A–F**). However, fecal microbiomes can be well separated into three groups according to Bray–Curtis (*r* ^2^ = 0.2240, *P* = 0.001; **Figure 3A**), weighted UniFrac (*r* ^2^ = 0.159, *P* = 0.001; **Figure 3B**), and unweighted UniFrac (*r* ^2^ = 0.118, *P* = 0.001; **Figure 3C**) analyses. Moreover, a Venn diagram comparison showed that the number of OTUs in the control, CPZ, and CPZ + Ven groups was 690, 665, and 714, respectively (**Figure 3D**). The number of shared OTUs among the three groups was 458, while 147, 85, and 113 OTUs were unique to the control, CPZ, and CPZ + Ven groups, respectively.

**Figure 4:**
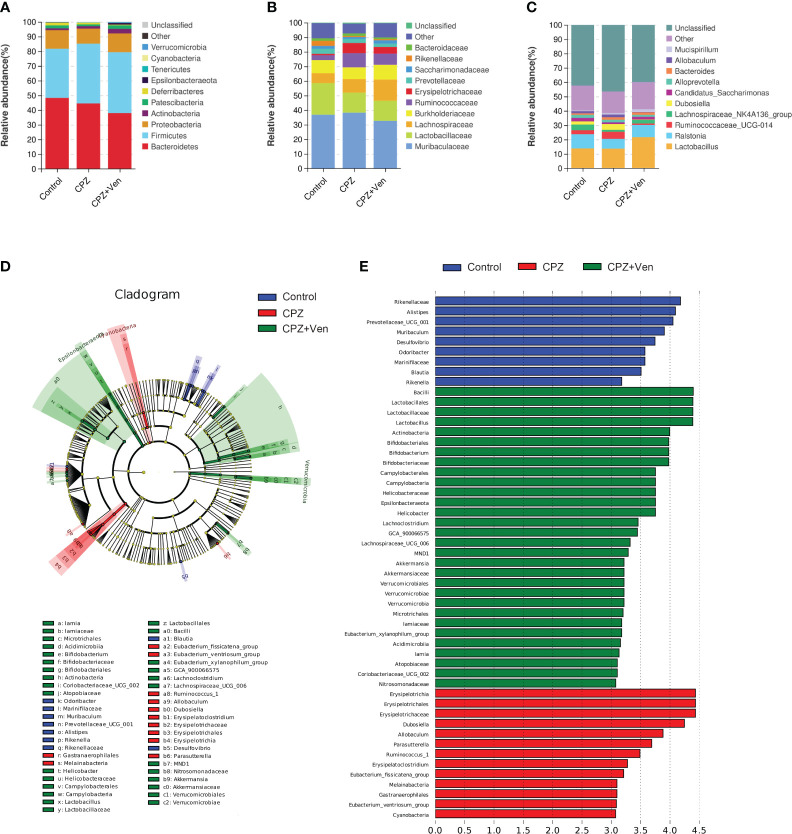
The comparison of taxonomic composition among control, cuprizone (CPZ), and CPZ + Ven groups. Community bar plot analysis showing the relative abundance of sequences at the **(A)** phylum, **(B)** family, and **(C)** genus levels. **(D)** The taxonomic cladogram shows the bacterial taxa enriched in control (blue dots), CPZ (red dots), and CPZ + Ven groups (green dots). **(E)** The LDA discriminant bar chart shows the microbial taxa with significant differences in the control (blue), CPZ (red), and CPZ + Ven groups (green). Larger LDA scores represent a greater effect of species abundance on the different effects. Only taxa with an LDA score >3.0 are shown in the figure.

As shown in **Figure 4A**, *Bacteroidetes*, *Firmicutes*, *Proteobacteria*, and *Actinobacteria* were the most abundant bacterial phyla distributed in the three groups. At the family level, *Muribaculaceae*, *Lactobacillaceae*, *Lachnospiraceae*, *Burkholderiaceae*, *Ruminococcaceae*, *Erysipelotrichaceae*, *Prevotellaceae*, *Saccharimonadaceae*, and *Rikenellaceae* dominated in the three treatment groups of mice (**Figure 4B**). At the genus level, *Lactobacillus*, *Ralstonia*, *Lachnospiraceae_NK4A136_group*, *Ruminococcaceae_UCG-014*, *Dubosiella*, *Candidatu_saccharimon*, *Alloprevotella*, *Bacteroides*, *Allobaculum*, and *Mucispirillum* were mainly distributed in the three groups (**Figure 4C**).

To distinguish the major taxa and to reveal changes in specific bacteria and the whole gut microbial community among the three groups, LEfSe analysis was performed. As shown in **Figures 4D, E**; **Supplementary Table S1**, the relative abundance of six family (such as *Leuconostocaceae*, *Rikenellaceae*, and *Clostridiaceae_1*) and 11 genera (such as *Prevotellaceae_UCG_001, Ileibacterium*, and *Blautia*) was enriched in the control group; family *Erysipelotrichaceae, Mycoplasmataceae*, and *Family_XIII* and 17 genera (such as *Ruminococcus_1*, *Candidatus_Stoquefichus*, and *Lachnospiraceae_FCS020_group*) were enriched in the CPZ group; phylum *Spirochaetes*, *Verrucomicrobia*, and *Epsilonbacteraeota*, 11 families (such as *Streptococcaceae*, *Akkermansiaceae*, and *Iamiaceae*), and 15 genera (such as *Streptococcus*, *Coriobacteriaceae_UCG_002*, and *UBA1819*) were enriched in the CPZ + Ven group.

### 3.3 Correlation between the gut microbiome composition and depressive-like behaviors

**Figure 5:**
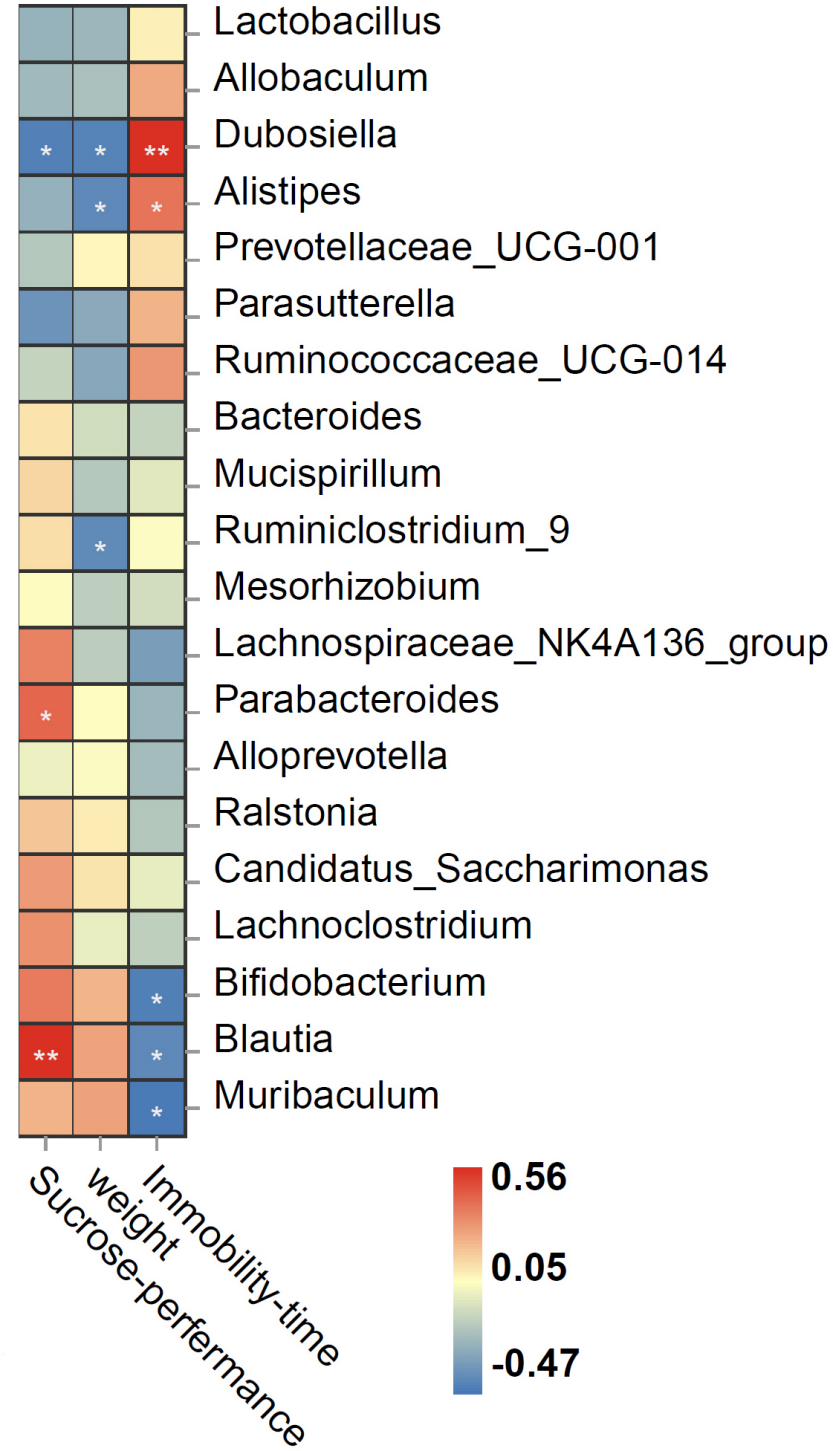
Spearman correlation heat map ranking the correlations between behavioral indices and bacterial abundance. The red and blue squares indicate positive and negative correlations, respectively, and the intensities of the colors are proportional to the degree of correlation. *P < 0.05; **P < 0.01.

Spearman correlation showed that the relative abundance of genus *Dubosiella* was negatively correlated, whereas those of *Blautia* and Parabacteroides were positively correlated with sucrose preference rate; the relative abundance of *Dubosiella*, *Alistipes*, and Ruminiclostridium_9 was negatively correlated with body weight; *Dubosiella* and *Alistipes* were positively correlated, whereas *Bifidobacterium*, *Blautia*, and *Muribaculum* were negatively correlated with immobility time (**Figure 5**).

## 4 Discussion

Depression poses a huge economic and medical burden to society and individuals due to its high mortality and disability rates, and investigation for the pathogenesis of depression is still urgently needed (51, 52). The gut microbiota, an integral part of the body, performs basic immune, metabolic, structural, and neurological functions and can even be called one of the “organs” of the body (53, 54). Multiple studies have shown that the gut flora can communicate with the brain and *vice versa* and is associated with the central nervous system through the microbiota–gut–brain axis (MGBA), thus affecting neurological and behavioral functions. Based on current data, depression is inextricably linked to altered gut microbiota, and regulation of the MGBA is emerging as a new target for the treatment of depression (55, 56).

CPZ acts specifically on oligodendrocytes in the central nervous system, causing cell death in the oligodendrocyte line and further leading to extensive demyelination of the corpus callosum, cortex, and hippocampus (57). This pattern of cell death is similar to the white matter abnormalities detected by neuroimaging in patients with demyelinating diseases (58). Although CPZ-induced demyelination is often used as a multiple sclerosis (MS) model, it also showed depressive-like behaviors, such as decreased autonomy and reduced spatial exploration (59). In fact, more than half of patients with MS exhibit depression comorbidities, indicating that both MDD and MS may have some common biological abnormalities simultaneously (e.g., deficits of myelin and excessive neuroinflammation) (60–62). Consistent with these results (47, 63), the present study found that 5 weeks of CPZ feeding induced a decrease of the sucrose preference rate and an increase in immobility time in TST but did not influence the locomotion in mice. These results indicate that the CPZ model might serve as a model that can simulate demyelination injury in MDD. Moreover, we found that Ven treatment ameliorated depressive-like behaviors induced by CPZ. Ven is one of the widely used antidepressants in clinical practices. Its neuroprotective and anti-inflammatory effects have been reported previously (64). A recent study found that Ven improves motor performance and sensitivity to a cold stimulus and ameliorates the deficit of superoxide dismutase activity in the mouse model of CPZ-induced demyelination. Ven alongside improves depressive-like behavior related to demyelination, indicating that Ven may have a potential improvement effect on MDD with abnormal white matter.

Multiple evidences elucidate that the abnormality in gut microbiota plays a role in the pathogenesis of MDD. The effect of CPZ on the gut microbiota of mice has been reported. Previous studies found that there was a significant difference in β-diversity, but not in α-diversity, after 6 weeks of CPZ treatment (46, 65). Similarly, the present study found that there was a significant difference in comparative β-diversity, but not in α-diversity, among the control, CPZ, and CPZ + Ven groups, indicating that Ven can restore the CPZ-induced abnormalities of β-diversity in the gut microbiota. Due to β-diversity reflecting the degree of change in community composition or the degree of community differentiation, we speculate that Ven has a more significant impact on the composition of microbial communities. Interestingly, a previous study found that Ven reduced the richness of microbial communities but did not affect their evenness in alpha diversity analyses, whereas the β-diversity of fecal microbial communities from mice receiving Ven was higher than that of the control samples (42), and this effect might be related to its regulation of the 5-HT, 5-hydroxyindoleacetic acid (5-HIAA), and glutamate (Glu) levels (66). The effect of Ven on 5-HT, 5-HIAA, and Glu of CPZ-treated mice still needs further observation. Furthermore, Moles et al. reported that the Shannon index was increased at the beginning and progressively decreased from week 2 during CPZ feeding, whereas microbial richness seems to increase during the CPZ intake (37). It is well known that C57BL/6 mouse fed with 0.2% (w/w) cuprizone induced the demyelination process, which can be detectable after 3 weeks of treatment and was complete at week 5. Then, the remyelinated axons appeared between weeks 5 and 6 (67). It suggests that the impact of CPZ on microbial α-diversity is inconsistent at different stages of demyelination/remyelination and the abnormal β-diversity persists during demyelination in CPZ-treated mice. Given that this study only observed diversity after 5 weeks of demyelination, the impact of different CPZ intervention durations on microbial diversity remains to be studied.

LEfSe analysis was performed to reveal changes in specific bacteria among the three groups. The results showed that 17 genera were enriched in the CPZ group, which were related to metabolism and inflammation—for example, *Lachnospiraceae_FCS020_group* was increased in patients with chronic kidney disease (68), *Ruminococcus_1* was positively correlated with the expression levels of proinflammatory cytokines tumor necrosis factor α (TNF-α) and interleukin-1β (IL-1β) in the offspring brain of maternal sleep deprivation rats (69), whereas *Candidatus_Stoquefichus* was negatively correlated with both TNF-α and IL-4 concentration during postpartum uterine recovery in mice (70). Meanwhile, 15 genera (such as *Lactobacillus* and *Bifidobacterium*) were enriched in the CPZ + Ven group. *Lactobacillus* and *Bifidobacterium* are probiotics (71), indicating that the regulatory effect of Ven on the gut microbiota of CPZ-treated mice might be related to upregulating the relative abundance of probiotics. However, the relationship between these changed bacteria and the efficacy of Ven is still unclear, and its mechanism of action needs further clarification.

Furthermore, genera *Dubosiella* was enriched in the CPZ group, and its relative abundance was negatively correlated with sucrose preference rate and was positively correlated with immobility time. A previous study indicated that *Dubosiella* is related to SCFAs produced, and *Dubosiella newyorkensis*, a strain of *Dubosiella*, possessed a strong effect of increasing superoxide dismutase (SOD) in aged mice (72). Therefore, the effect of CPZ on demyelination and depressive-like behaviors may be related to the excessive oxidative stress and inflammation. Besides this, genus *Bifidobacterium* was enriched in the CPZ + Ven group, and its relative abundance was negatively correlated with immobility time. Genera *Alistipes*, *Blautia*, and *Muribaculum* were enriched in the control group, and their relative abundance was positively or negatively correlated with depressive-like behaviors. Given that *Bifidobacterium* and *Blautia* are well-known probiotics (73, 74) and the decrease of *Muribaculum* was related to acute sleep deprivation-induced psychological defects (75), these results indicate that CPZ may lead to a depressive-like behavior by reducing the beneficial bacteria, and *Bifidobacterium* might be the key bacteria involved in the antidepressant effect of Ven. Nevertheless, it is a limitation in such a way that we did not clarify which species are affected by CPZ or Ven—for example, there is contrasting evidence reported that *Alistipes* may have protective effects against cancer immunotherapy (76), liver fibrosis (77), and cardiovascular disease (78). However, *Alistipes* is also pathogenic in depression (79) and colorectal cancer (80). Since genus *Alistipes* consists of 13 species, the species that causes this functional difference may be more critical. Similarly, a previous study found that *Lactobacillus rhamnosus* reduces stress-induced anxiety- and depression-related behaviors by regulating the central GABA receptor expression (81). In contrast, another study found that *Lactobacillus reuteri* and *Lactobacillus intestinalis* induces depression phenotypes in antibiotic-treated mice (82). Therefore, different species of the same genus have inconsistent or even opposite functions, and future research needs to be precise to the level of species.

The individual differences in animal behavior are also worth exploring—for example, some mice were able to develop a sucrose preference in the CPZ group, while individual mice in the control group did not develop the sucrose preference, although there is a significant difference in sucrose preference between the two groups. These differences might be attributed to the plasticity or flexibility of individual behavior, which was related to a series of factors such as gene modification, neural circuit function, and stress regulation (83, 84). Meanwhile, behavioral detection methods, such as sucrose preference and tail suspension experiments, are both methods to detect a depressive-like behavior and forms to cope with stress in mice (85). Further research is needed to investigate the effects of these stresses on brain function and myelin integrity.

In conclusion, the present study suggests that CPZ induces disruption of the gut microbiota in mice, including characteristic changes in community diversity, taxon abundance, and depressive-like behaviors. Ven ameliorates CPZ-induced depressive-like behaviors and upregulates the relative abundance of probiotics, such as *Bifidobacterium* and *Lactobacillus*. However, the specific mechanism by which Ven affects the microbiota and the correlation between microbiota changes and myelin repair still needs to be elucidated. Further observation of the influence of Ven on peripheral and brain monoamine neurotransmitters, fatty acids, inflammatory response, and oxidative stress levels will help explain this effect. Nonetheless, the mice were group-housed in cages with unrestricted access to standard powdered rodent chow that contained CPZ (0.2% w/w) in the present study, which was performed according to previous studies. We cannot be sure that all the animals inside the cage have submitted to the same levels of CPZ. On the other hand, although single-cage feeding can accurately evaluate the intake of CPZ, long-term single-cage feeding is also a strong negative stress, and it is difficult to rule out the impact on mouse behavior. It is worth noting that the effects of single-cage and group-housed feeding of CPZ on mouse demyelination and behavior need to be further explored. Finally, several shortcomings of this study need to be pointed out. Firstly, the impact of different doses and duration of Ven on the microbiota of normal or CPZ-treated mice is not yet clear. Secondly, we did not observe whether the effect of Ven on the microbiota was related to its myelin-protective effect. Moreover, the molecular mechanisms by which Ven regulates the gut microbiota is still unclear. Further investigations need to observe the impact of Ven on microbial communities at the species level and further explore its potential mechanisms of action.

## Data availability statement

The original contributions presented in the study are included in the article/**Supplementary Material**. Further inquiries can be directed to the corresponding authors.

## Ethics statement

The animal study was approved by the Animal Experimental Welfare and Ethical Inspection of Air Force Medical University. The study was conducted in accordance with the local legislation and institutional requirements.

## Author contributions

NT: Conceptualization, Formal Analysis, Funding acquisition, Project administration, Supervision, Writing – review & editing, Writing – original draft. CD: Conceptualization, Funding acquisition, Supervision, Writing – review & editing. TZ: Data curation, Formal Analysis, Methodology, Writing – original draft. CF: Data curation, Formal Analysis, Supervision, Writing – review & editing. QS: Data curation, Formal Analysis, Investigation, Writing – review & editing. ZC: Data curation, Formal Analysis, Writing – review & editing. XS: Data curation, Formal Analysis, Writing – review & editing. YL: Data curation, Formal Analysis, Writing – review & editing. GD: Data curation, Formal Analysis, Writing – review & editing. XZ: Conceptualization, Project administration, Supervision, Writing – review & editing.

## Supplementary material

Supplementary Table 1 Comparison of differences in microbial communities at different levels among three groups.
